# Relation Between Thermal Analysis, Phase Composition and Structure of Polyurethane Adhesives for Application in Wooden Structural Joints

**DOI:** 10.3390/polym18111396

**Published:** 2026-06-04

**Authors:** Magdalena Szumera, Paweł Rutkowski, Anna Berezicka, Marcin Gajek, Bartosz Handke, Piotr Jeleń, Konrad Kwiecień, Arkadiusz Kwiecień, Klaudia Śliwa-Wieczorek

**Affiliations:** 1 Department of Ceramics and Refractories, Faculty of Materials Science and Ceramics, AGH University of Krakow, al. A. Mickiewicza 30, 30-059 Krakow, Poland; berezicka@agh.edu.pl (A.B.); mgajek@agh.edu.pl (M.G.); 2 Department of Silicate Chemistry and Macromolecular Compounds, Faculty of Materials Science and Ceramics, AGH University of Krakow, al. A. Mickiewicza 30, 30-059 Krakow, Poland; bhandke@agh.edu.pl (B.H.); pjelen@agh.edu.pl (P.J.); 3 Department of Biomaterials and Composites, Faculty of Materials Science and Ceramics, AGH University of Krakow, al. A. Mickiewicza 30, 30-059 Krakow, Poland; kkwiecien@agh.edu.pl; 4 Department of Structural Mechanics and Material Mechanics, Faculty of Civil Engineering, Cracow University of Technology, Warszawska 24 Street, 31-155 Krakow, Poland; arkadiusz.kwiecien@pk.edu.pl; 5 Department of Bridge, Metal and Wooden Structures, Faculty of Civil Engineering, Cracow University of Technology, Warszawska 24 Street, 31-155 Krakow, Poland; klaudia.sliwa-wieczorek@pk.edu.pl

**Keywords:** polyurethane adhesives, DSC-TGA-QMS methods, HSM, dilatometry, FT-IR spectroscopy, X-ray analysis

## Abstract

Due to the possibility of damage from earthquakes, vibrations, humidity, and the degradation of wooden joints, there is growing interest in new polyurethane adhesives for wooden structures. These adhesives often have two or more purposes in such structures. Such purposes include connection strength, resistance to environmental conditions at the point of application, and behaviour during fire or under vibration. Some fundamental data on the application of materials exhibiting these properties concern their adhesive thermal stability. This paper focuses on the thermal stability of a new blend of flexible and rigid polyurethanes and its correlation with the structural properties of the material. The new polyurethanes were investigated using hot-stage microscopy for thermal stability of shape, and the results were correlated with DSC, thermogravimetry, and evolved-gas analyses. The experiments showed that it is essential to use primary research methods, including FTIR, XRD, DSC-TG-QMS, and HSM, to identify and characterise new polyurethane adhesives. These research methods are crucial for understanding the properties and potential applications of these materials and providing deeper insight into the subject. The tested polyurethane adhesives, new materials for construction, meet strict ecological requirements and are suitable for patching both small and large wooden structures, as well as for other construction applications, such as insulation and soundproofing.

## 1. Introduction

Polyurethane adhesives, with their versatile bonding capabilities, have found wide application across various industries. These adhesives are available in rigid and flexible forms, each with distinct properties and applications shaped by their structural characteristics; it is essential to select the appropriate adhesive for a specific application. Polyurethane adhesives used in wooden structural joints must simultaneously provide mechanical integrity, dimensional stability, and resistance to thermal degradation under service and fire-exposure conditions. Although polyurethane systems for timber engineering (mainly using rigid one-component polyurethane—PUR, commercially available LOCTITE^®^ PUR of Henkel) have been extensively studied, the relationship between filler composition, evolved-gas generation, and thermally induced dimensional instability remains insufficiently understood, particularly for hybrid flexible–rigid polyurethane systems (those using two-component flexible polyurethane—FPU). The two-component polyurethanes in this group exhibit flexible properties and varying rigidity and are the main focus of this paper. Previous studies on polyurethane wood adhesives have primarily examined the effects of polymer network architecture, residual NCO content, curing kinetics, creep resistance, and conventional fillers on mechanical or thermomechanical performance [1,2,3]. Inorganic and organic fillers, such as calcium carbonate, silica, polyamide particles, SAN dispersions, and polyurea modifiers, have been reported to improve selected mechanical, rheological, and thermal properties of polyurethane adhesive systems [3,4,5,6,7,8,9,10,11]. However, systematic studies concerning flexible–rigid polyurethane systems containing sodium aluminosilicate hydrate (N-A-S-H) combined with flame-retardant mineral fillers remain very scarce in the available literature. In particular, the influence of N-A-S-H-derived water release, phase composition, and interactions between hydrated aluminosilicate phases and polyurethane degradation processes on the thermal stability and dimensional behaviour of polyurethane adhesive systems has not yet been comprehensively investigated [12,13,14,15]. This aspect is particularly important because N-A-S-H phases contain both physically retained and structurally bound water, which may influence local gas evolution, transient swelling behaviour, heat-transfer conditions, and diffusion pathways during polyurethane decomposition at elevated temperatures. Moreover, previous studies have rarely combined structural, phase, thermo-analytical, evolved-gas, and direct shape-stability analyses within a single experimental methodology, particularly for polyurethane systems intended for bonded wooden structural joints [1,16,17,18,19].

Rigid one-component polyurethane adhesives, used mostly in wooden structures as a very thin layer of PUR (thinner than 0.2 mm), are characterised by relatively high stiffness and a tendency to brittle failure. To avoid these drawbacks, two-component polyurethanes called FPUs started to be used. They can form adhesive layers up to 30 mm thick and are thus more flexible due to their greater thickness, chemical composition, and fillers. Various required properties, such as stiffness, ultimate elongation, and strength, are designed into those FPU formulations in ways known only to the makers. They can be made with different levels of rigidity, ranging from high to low. Rigid PUR adhesives are characterised by high isocyanate-to-polyol ratios, which result in dense network structures. This composition directly contributes to their high tensile and compressive strength, making them well-suited for heavy-duty applications requiring robust bonding. One of the most notable advantages of rigid polyurethane adhesives is their dimensional stability. They can maintain their shape under load and during temperature fluctuations, ensuring a consistent bond even in challenging environmental conditions [20,21]. Additionally, rigid polyurethanes exhibit thermal resistance, offering good protection against heat and chemical exposure. However, a fundamental limitation of rigid polyurethane adhesives is their low flexibility. Due to their stiffness, these adhesives may crack under stress or impact, limiting their use in applications involving dynamic forces or movement. One typical application for rigid polyurethane adhesives is structural bonding, where strong and stable adhesion is paramount. They are frequently used in industries such as automotive, furniture assembly, and construction to bond components such as panels and frames, providing long-lasting, reliable bonds in load-bearing structures [22].

On the other hand, flexible polyurethane FPU adhesives are formulated with a higher proportion of flexible polyols, resulting in lower density and greater elasticity. This composition allows for significantly greater flexibility, enabling these adhesives to accommodate movement and deformation without cracking. This makes flexible polyurethanes ideal for dynamic applications where materials are subject to movement or vibration. Flexible polyurethane FPU adhesives also exhibit good adhesion to a wide range of substrates, including similar and dissimilar materials, enhancing their versatility across various industries. While they maintain decent tensile strength, they generally do not offer the same level of load-bearing capacity as rigid polyurethanes. However, their ability to absorb shock and vibrations makes them particularly effective in environments where impact resistance is a priority [23,24]. Flexible polyurethane adhesives are commonly used in applications such as automotive interiors, where components must remain securely bonded while accommodating movement and vibration. They are also used in electronics, footwear, and other products that require a degree of flexibility in adhesive bonds [25].

Polyurethane adhesives, with their unique properties such as strong adhesion, flexibility, and resistance to moisture and chemicals, have found widespread application in wood construction. These adhesives are primarily used in wood construction to bond wood laminates, engineered wood products, and composite materials. The strong adhesion provided by polyurethane adhesives ensures durable joints that maintain the structural integrity of wood constructions, such as flooring, furniture, and cross-laminated timber (CLT) [26]. These adhesives form resilient bonds that can endure the stresses of load-bearing applications, making them particularly valuable in structural applications where wood products must remain secure over time.

Polyurethanes are also fundamental in producing composite wood products, such as cross-laminated timber (CLT), an engineered wood product known for its strength and dimensional stability. Incorporating polyurethanes into the CLT manufacturing process improves the performance of these materials, making them more robust, more durable, and better suited for large-scale construction projects. The growing popularity of CLT in the construction industry, particularly in tall wood buildings, demonstrates the increasing reliance on polyurethane products in advanced wood construction techniques. However, the versatility of polyurethanes is not limited to new construction. In repair and renovation projects, these materials play a significant role. For instance, in the repair process, polyurethane-based materials can be used to fill gaps or reinforce the integrity of wood that has decayed or sustained structural damage over time. This cost-effective, efficient method for restoring wooden buildings, furniture, or other wooden elements with low-VOC polyurethane prolongs their lifespan. It reduces the need for complete replacement, making polyurethane a valuable asset in the construction industry [27,28].

However, to fully realise the benefits of polyurethanes in wood structures, specific requirements must be met for practical use. One such requirement is adhesion performance. Polyurethane adhesives must bond effectively to various types of wood and other construction materials, ensuring that the joints they form remain secure and durable over time. Furthermore, given wood’s natural tendency to absorb moisture, polyurethane products must exhibit strong moisture resistance. This helps prevent swelling, warping, or degradation of the wood, which could compromise the integrity of the construction [29]. Another essential requirement is the flexibility of polyurethane formulations. Since wood expands and shrinks with changes in temperature and humidity, flexible polyurethanes are necessary to accommodate this natural movement without causing cracks or other damage to the wood. Environmental considerations must also be taken into account in light of growing ecological concerns [30,31]. Many polyurethane products contain volatile organic compounds (VOCs), which can negatively impact the environment and health. As a result, there is a growing preference for low-VOC or environmentally friendly polyurethane options, particularly in residential construction, where occupants’ health and safety are paramount. Compatibility with finishing products is also essential when using polyurethane adhesives and coatings in wood construction [1,32,33]. Looking to the future, further research and development in polyurethane adhesives could yield even more advanced formulations that meet these requirements and open new possibilities for their use in wood construction.

Manufacturing PU adhesives typically involves reacting polyols with isocyanates, enabling highly customizable properties tailored to specific applications. Polyols provide flexibility, while isocyanates impart rigidity, creating a balance between elasticity and strength that makes PU adhesives suitable for a range of applications. Recent trends in the manufacturing of PU adhesives focus on sustainability, advanced material properties, and intelligent adhesive technologies [33,34]. One significant development in PU adhesive manufacturing is the shift toward eco-friendly formulations. Increased environmental regulations and the demand for sustainable products drive this shift. Researchers are exploring bio-based polyurethanes derived from plant-based polyols, such as vegetable oils, to reduce the carbon footprint of PU production [35,36].

Additionally, non-isocyanate polyurethane (NIPU) adhesives are being developed in order to eliminate toxic isocyanates, improving safety and environmental sustainability [37,38,39]. Another area of innovation is the incorporation of nanomaterial additives. Nanomaterials, such as nanoclays, graphene oxide, and carbon nanotubes, are added to improve the mechanical, thermal, and adhesive properties of PU adhesives. These nanomaterials enhance the adhesives’ thermal stability, mechanical strength, and moisture resistance, making their use suitable in harsh environments [40,41]. In parallel, innovative adhesives have gained traction, particularly self-healing PU adhesives that can autonomously repair minor damage, extending the service life of bonded components [42]. Additionally, stimuli-responsive PU adhesives that respond to external triggers such as heat, light, or moisture are being developed for specialised applications in electronics and biomedical devices [43,44].

The thermal examination of PU adhesives considers thermal stability. Various thermal analysis techniques are employed to evaluate the performance of PU adhesives under thermal stress, with particular attention to degradation temperature, phase transitions, and temperature-dependent mechanical properties. Thermogravimetric Analysis (TGA) is a technique commonly used to assess the thermal stability of PU adhesives by measuring weight loss as the material is heated. PU adhesives generally degrade in two stages: first, the degradation of soft polyol segments, followed by that of hard isocyanate segments. This data provides insights into the adhesive’s ability to withstand high temperatures [45]. Differential Scanning Calorimetry (DSC) is another crucial technique, measuring heat flow during phase transitions, including the glass transition temperature (T_g_), melting points, and crystallisation. The T_g_ determines how the material performs at different temperatures [46,47]. Recent trends in thermal examination [16,48] focus on developing high-temperature adhesives and improving thermal conductivity. PU adhesives with modified formulations are designed to withstand higher operating temperatures, making them suitable for more extreme environments. Furthermore, improved thermal conductivity is an area of growing research, especially in the electronics industry, where efficient heat dissipation is crucial. Additives such as boron nitride and graphene are incorporated into PU adhesives to enhance their thermal management properties [49,50].

As mentioned in the paragraph above, polyurethane is used as an adhesive or foam with various modifiers. The most common are carbonate fillers, which improve the material’s mechanical wear resistance [4] and can influence its behaviour as a fire-resistant material [5]. Also, aluminium hydroxide is often used as an additive to various polymers. Its function is to decrease a flame’s temperature. This occurs due to decomposition and the release of water vapour, which dilutes polymer gases at higher temperatures [6]. There are also other additives, such as titanium oxide, aluminium oxide, and various spinels, which, on the one hand, act as flame retardants and, on the other hand, improve PU ageing resistance [7]. The BaSO_4_ filler, used with various polymers, exhibits supercrystallinity and is thermally stable at very high temperatures [8]. However, it causes material agglomeration. It is stable at least up to 1200 °C [9], but in a carbon monoxide-rich atmosphere, this temperature limit lowers to 850–1000 °C, which is sufficient for PU applications [10]. Such modifiers should not be mixed with carbonate. Used separately, this filler improves polymer thermal stability [11] and mechanical properties. The new N-A-S-H phase (Sodium Calcium aluminium Silicon Oxide Hydrate) [12], which contains a high amount of water, was used in the present work. According to Hou’s [12] investigation, large amounts of water can be released at higher temperatures, decreasing flame temperature. The C-S-H-like phase can also improve the glue’s mechanical and adhesive properties below the PU thermal-stability temperature. So, these additives will influence the thermal stability and behaviour of flexible and rigid PU at elevated temperatures.

Considering the papers in the literature mentioned above, which report correlations between polyurethane’s adhesive structure and functional properties, the main objective of this article was to thoroughly investigate the structural features and thermal characteristics of selected polyurethane adhesives. The first objective of the research was to examine hybrid flexible two-component polyurethane systems (FPU) of a wide range of stiffness (from rigid to flexible) with respect to their structure (FT-IR) and crystalline phase composition (XRD). Due to their intended innovative application in bonded wooden structural joints, the investigated polyurethane mixtures were additionally subjected to complementary thermal analyses to evaluate their thermal degradation behaviour and dimensional stability during heating. Compared with our previous studies [30], the present work extends the thermal characterisation by incorporating evolved-gas analysis at elevated temperatures, using DSC-TG-QMS measurements together with hot-stage microscopy (HSM) analysis of thermally induced shape stability. The combined application of FTIR, XRD, DSC-TG-QMS, and HSM enabled the analysis of relationships among filler composition, evolved gases, thermal degradation behaviour, and the dimensional stability of the investigated polyurethane systems. Particular attention was devoted to polyurethane compositions containing sodium aluminosilicate hydrate (N-A-S-H) and mineral flame-retardant fillers, the combined structural and thermo-analytical characterisation of which remains limited in the available literature. Therefore, the novelty of the present work lies in both the investigation of N-A-S-H-containing mineral-modified flexible–rigid polyurethane systems and an integrated thermal–structural correlation methodology that combines FTIR structural analysis, XRD phase identification, DSC-TG-QMS evolved-gas measurements, and hot-stage microscopy observations. This combined analytical approach enabled direct correlation of filler phase composition, water-release mechanisms associated with N-A-S-H and gibbsite (Al(OH)_3_) decomposition [12,14,15,51], gaseous degradation products, thermal decomposition pathways [17,18,19], and thermally induced dimensional instability during heating. The proposed methodology also enabled interpretation of how individual mineral phases influence gas-diffusion behaviour, transient shape stability, swelling phenomena, and the high-temperature structural integrity of polyurethane systems under thermally induced degradation conditions. In contrast to previous studies focused mainly on conventional thermal analysis or adhesive mechanical performance, the present work combines phase-resolved XRD analysis, evolved-gas identification, and direct observation of thermally induced shape changes within a single analytical framework, allowing the degradation behaviour of multicomponent polyurethane systems to be interpreted in relation to both polymer chemistry and filler-phase transformations. The investigated FPU materials were prepared according to the patented formulations described in Ref. [52], and the patent owners agreed to the scientific characterisation and publication of these materials.

## 2. Materials and Methods

### 2.1. Materials

The five commercially available polyurethane adhesives investigated, distributed by FlexAndRobust Systems (Cracow, Poland), belong to the P category of two-component polyurethanes (FPU) and are sold under the commercial names F&R PM, F&R PS, F&R PST, F&R PTS, and F&R PT. They comprise certain patented technologies, namely, PolyUrethane Flexible Joints (PUFJ) and Fiber Reinforced PolyUrethanes (FRPU), which are protected under doctrines of “know-how” and Intellectual Property Rights (IPR); thus, their chemical formulations are not available, and the company characterizes them in publications only by their mechanical properties. Detailed mechanical properties of these FPU materials, such as the rigidity ratio described by Young’s modulus, were obtained from tests. The results from the research were described in [53,54,55]. Additional information on the material properties of these FPU can be found in [56,57]. These adhesives have been successfully utilised to repair concrete surfaces and strengthen both concrete beams and masonry structures due to their hyperelastic properties [58]. The five new flexible polyurethanes (FPU) of various flexibility were prepared for measurement. These FPU materials, protected by IPR, were developed [52] to offer specific properties suitable for various industrial applications, with the primary areas of focus being flexibility and elasticity and their protective properties. Below is a detailed description of the materials and their respective component ratios (provided only by the distributor, due to IPR). For each polyurethane formulation, three independently prepared specimens were used in the experimental procedure, in order to verify the repeatability of the results and confirm the homogeneity of the investigated polyurethane adhesive systems.

The first sample, F&R PS (short name: PS), is a solvent-free, two-component polyurethane adhesive. This material is notable for its flexibility, a quality which makes it ideal for forming flexible joints and protective coatings. Its solvent-free nature enhances its environmental benefits, as it does not rely on harmful chemicals for its functionality. The component ratio, with A = 100 and B = 11, reflects the balance between components required to achieve the desired adhesive and flexibility. This formulation is well-suited for applications requiring movement or deformation, providing a durable yet flexible bond. The second sample, F&R PST (short name: PST), is also a two-component polyurethane-based adhesive. Like the PS, it exhibits flexibility, making it suitable for joints and protective coatings. However, the component ratio for PST differs slightly: A = 100 and B = 15. This variation in component proportion subtly alters the material’s characteristics, potentially enhancing its flexibility and adhesion for more demanding applications. The PST was designed with a specific focus on flexibility, allowing it to adapt to a wide range of movements without compromising the integrity of the bonded surfaces. F&R PT (abbreviated as PT) is another crucial material in this study. It is a solvent-free, two-component polyurethane adhesive that hardens to a hard-elastic state. Unlike the previous two samples, PT is formulated to provide a stiffer yet flexible bond, ideal for applications requiring both rigidity and elasticity. The component ratio for PT is significantly higher, with A = 100 and B = 52, contributing to its stable final state. This composition is particularly well-suited for applications requiring a balance between flexibility and structural integrity, such as protective coatings that must withstand environmental and mechanical stresses. The fourth material, F&R PTS (abbreviated as PTS), is similar in base formulation to a two-component polyurethane adhesive, but its permanent elasticity sets it apart. This material was developed specifically for flexible joints and protective coatings that require long-term flexibility. The component ratio for PTS is A = 100 and B = 15, indicating a formulation similar to PST and one focused on maintaining elasticity over time. This characteristic makes PTS a valuable option for applications where joints or surfaces must remain flexible throughout their service life, even under continuous stress or environmental exposure. Finally, the fifth sample, F&R PM (short name: PM), is a high-resilience, two-component polyurethane adhesive. Like other materials, PM aims to create flexible joints and protective coatings, but its resilience and durability are its defining characteristics. Again, the component ratio is A = 100 and B = 15, suggesting a base formulation similar to PST and PTS. However, PM is distinguished by its ability to maintain performance under high stress and repeated use, making it ideal for applications requiring both flexibility and long-term resilience, such as heavy-duty industrial settings.

The five FPU samples—PS, PST, PT, PTS, and PM—were designed for specific applications, particularly flexible joints and protective coatings. Their unique component ratios and material properties enable them to perform in environments that require critical flexibility, elasticity, and durability. Through careful formulation and adherence to patented manufacturing processes, these materials demonstrate polyurethane’s versatility in modern adhesive applications.

### 2.2. Characterisation

The as-prepared materials were subjected to structural investigation by spectroscopic measurements. FT-IR measurements were performed using a Bruker Vertex 70v spectrometer with a diamond ATR attachment (Bruker Optik GmbH, Ettlingen, Germany); 128 scans at a resolution of 4 cm^−1^ were recorded in the 4000–400 cm^−1^ range. Each sample was measured three times, and the data were averaged. All the spectra were subjected to baseline treatment using the Bruker OPUS 7.2 software. Spectral deconvolutions were performed using Bruker OPUS 7.2 software following the procedure described in Ref. [59]. Because of the complex phase composition of manufactured F&RPU, the prepared adhesive samples were subjected to X-ray diffraction analysis using a X’Pert Pro MD (Malvern-Panalytical, Malvern, UK) powder diffractometer with Kα1 radiation from a Cu anode. The configuration was a standard Bragg–Brentano setup with a Ge (111) monochromator at the incident beam. All measurements were performed with a 0.016° step size over a 5–60° scanning range, with a 2 s measurement time per step. The pattern-fitting procedure for quantitative analysis and structural parameter determination was performed using the PANalytical HighScore Plus (version 3.0e, Malvern-Panalytical) software and the PDF-4+ 2024 (ICDD) database. XRD measurements were performed on three independently prepared specimens for each polyurethane system, and representative diffraction patterns were selected for phase identification and quantitative analysis.

In the next step, the structurally characterised and phase-characterised polyurethane materials were tested for thermal stability at elevated temperatures. For this purpose, two kinds of measurements were performed. The first part of the measurement was the thermal stability of the connected mass of FPU materials, which is directly derived from the material’s structure and composition. For this purpose, FPU was subjected to thermal analysis (DSC) as a function of temperature, and this was coupled with quadrupole mass spectrometry (QMS). The thermal and evolved-gas analyses (DSC-QMS) were performed using an STA 449 F3 coupled with a QMS 403d Aeolos from Netzsch (NETZSCH-Gerätebau GmbH, Selb, Germany). The measurements were made with 20 mL·min^−1^ air sample purging and 20 mL·min^−1^ device-protective argon gas. The test was performed at a heating rate of 10 °C·min^−1^, from RT to 1000 °C, in platinum crucibles. For each polyurethane adhesive, DSC-TG-QMS measurements were carried out on three independently prepared specimens to verify the repeatability of the thermal degradation behaviour, mass-loss steps, and evolved-gas profiles. The results presented in the manuscript are representative measurements that show consistent thermal characteristics. The second part of the thermal behaviour analysis of FPU, involving DSC-TGA-QMS and structural-phase studies, focused on the material’s shape stability as temperature increased. Misura^®^ HSM Expert System Solutions was used to investigate the flexible and rigid polyurethane samples to achieve this goal. This analysis was conducted under air-static conditions, at a heating rate of 10 °C·min^−1^, from RT to 1200 °C. The analysed sample had a diameter of 2 mm and a height of 3 mm. The characteristic recorded analysis points of material stability were determined, visualised, and correlated with structural and phase analysis and other thermal analysis measurements reported in this paper. Combining the results of the above-described studies, comprising both structural and thermal analysis, allowed us to explain the thermal stability of the new mixed flexible and rigid polyurethanes in bonded joint applications in wood constructions.

All structural, phase, and thermal analysis examinations were performed in the department and faculty central laboratories at the Faculty of Materials Science and Ceramics of AGH University of Krakow, Poland.

## 3. Results and Discussion

### 3.1. Spectroscopic Characterisation of FPU

**Figure 1:**
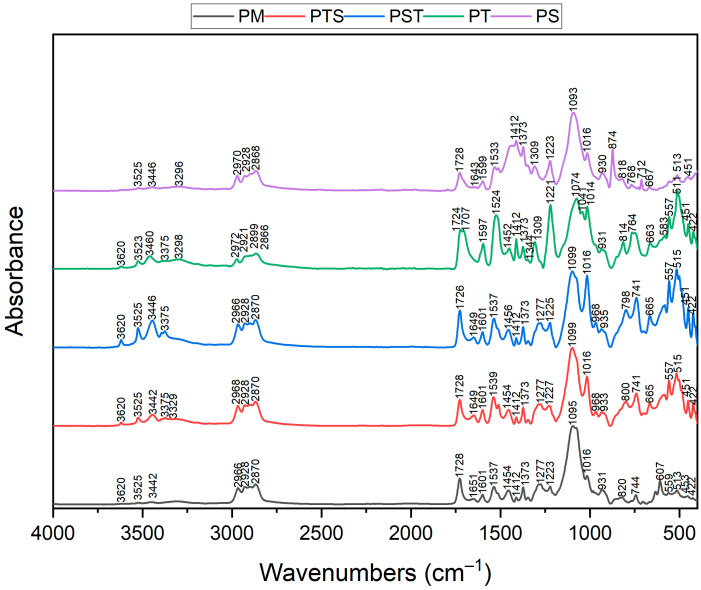
ATR-FTIR spectra of FPU.

The five different mixtures of flexible polyurethanes (coded as PS, PST, PT, PTS and PM) were subjected to FT-IR spectroscopy analysis. The results of recorded spectra for FPU samples, measured at room temperature, are presented in Figure 1. The analysed wavenumber ranges were assigned to their band groups and described in Table 1.

As shown in Figure 1, all tested materials exhibited typical polyurethane spectral features. The band at approx. 3300 cm^−1^ is responsible for N-H stretching vibration (H-bonded with oxygen), while the free N-H stretching bond is probably overlapping with O-H stretching vibrations arising from the additives. The 2900–2800 cm^−1^ bands can be related to the C-H stretching vibrations of CH_3_ and CH_2_ units from the polyurethane chain. As noted, there is no typical isocyanate band at approximately 2250 cm^−1^, indicating that residual free isocyanate groups are not detectable under the applied FTIR conditions. The carbonyl stretching vibration is visible at approximately 1728 cm^−1^. This can be connected with the free urethane vibrational mode. Also, a minimal shoulder at 1710 cm^−1^ can be attributed to hydrogen-bonded urethane carbonyls and/or associated carbonyl environments within the hard segments. All of the tested samples exhibit a band at approximately. 1650 cm^−1^. This vibration can be assigned to either the monodentate or bidentate H-bonded urea carbonyl stretching mode. The stretching vibration of the phenyl-derived C=C bond is present at approx. 1600 cm^−1^ and is visible for all tested materials. The C-N stretching and N-H bending vibration is visible at 1540 cm^−1^, while the C-N stretching from the amide III bond is visible at 1225 cm^−1^. The asymmetric and symmetric C-CH_3_ deformation vibrations are present at 1455 and 1375 cm^−1^, respectively. The C-H deformation vibrations of CH_2_-N, CH, and CH_2_ from the polyol can be observed at 1475, 1345, and 1300 cm^−1^, respectively. The C-O-C vibration of polyol is observed at 1095 cm^−1^. The above description in this paragraph was confirmed and described by Haseth [60]. All considerations have also been confirmed by other works [61,62,63]. The measured samples have inorganic fillers in their microstructure. The presence of additional bands in each recorded spectrum is manifest. All studied materials have bands at 3620, 3525, 3442, and 3375 cm^−1^. These can be attributed to the O-H stretching vibrations in interlayers, and the interlayers, the first and last two, respectively, in Al(OH)_3_–gibbsite [64,65]. The bands responsible for Al-O vibrations in the six-fold membered rings range from 780 to 420 cm^−1^ and overlap with O-H and FPU vibrations [64]. Also, all recorded spectra exhibit bands typical of LTA zeolite at approximately 1010, 665, and 556 cm^−1^, although they partially overlap with those of FPU and gibbsite. In the PS sample, calcite bands are also present at 1412, 784, and 712 cm^−1^. In the PM sample, the 1100 cm^−1^ band broadens, accompanied by the emergence of a new band at 607 cm^−1^. This is probably due to the presence of S-O bonds in the barite structure [66].

The carbonyl stretching region was analysed after the fitting process. Although seven mathematical components were used to reproduce the asymmetric C=O band shape, the individual bands were not interpreted as isolated molecular species, as shown in Table 2, Table 3, and the Supplementary Materials (Figures S1–S5). Instead, the fitted components were grouped into three spectral regions: strongly associated and/or urea-related carbonyls below 1690 cm^−1^, hydrogen-bonded urethane carbonyls between 1690 and 1720 cm^−1^, and free or weakly hydrogen-bonded urethane carbonyls between 1720 and 1750 cm^−1^. This approach provides a semi-quantitative comparison while avoiding overinterpretation of highly overlapping fitted components.

The highest fraction of associated carbonyl groups was observed for PT, with *F_assoc_* = 0.751 and *HBI* = 3.018, indicating the strongest contribution of hydrogen-bonded carbonyl environments. In contrast, PM exhibited the lowest carbonyl association, with *F_assoc_* = 0.432 and *HBI* = 0.760, and the highest contribution from free or weakly hydrogen-bonded carbonyls. PS, PST and PTS showed intermediate behaviour, suggesting moderate hard-segment association and hydrogen bonding within the polyurethane network. These parameters should be treated as comparative spectral indices rather than absolute concentrations of specific carbonyl species.

### 3.2. Phase XRD Characterisation of FPU

**Figure 2:**
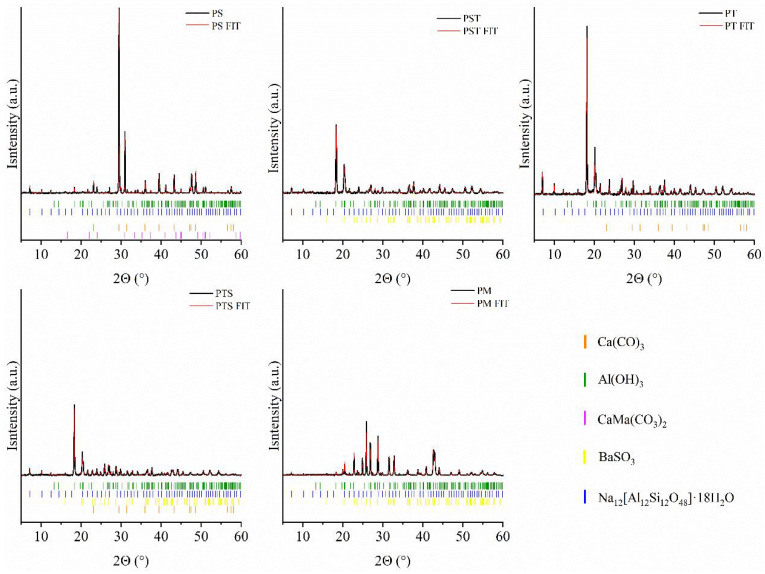
XRD phase composition of the crystalline part of FPU.

**Figure 3:**
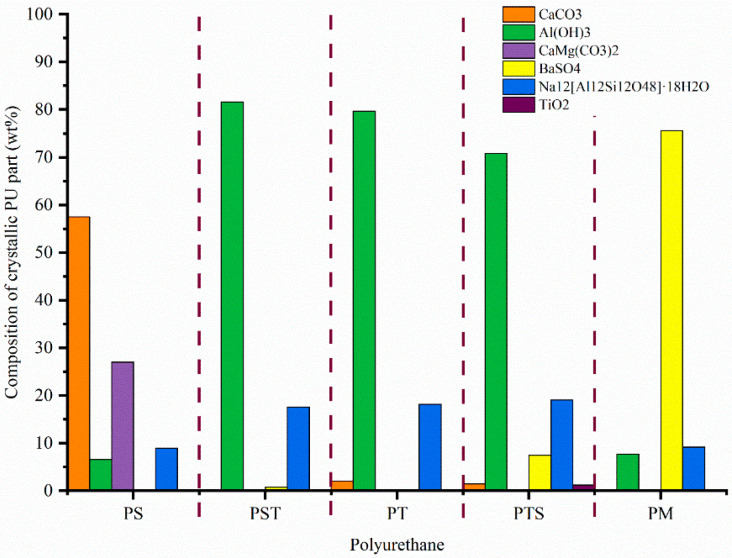
Crystalline phase composition of FPU.

XRD and Rietveld analysis were used to examine the crystalline phase of the mixture of flexible and rigid polyurethanes, providing qualitative and quantitative data on the added fillers. The XRD data are presented in Figure 2. The qualitative data and phase description are given in Figure 3 and collected in Table 4. The XRD analysis pertains only to the crystalline phase of FPU and does not account for the amount of FPU in the composition.

The results showed that FPUs have incorporated fillers that enhance mechanical properties and adhesive strength, improve anti-ageing properties, and act as flame retardants (Figure 2 and Figure 3). Each polyurethane contains 3–5 crystalline phases at varying concentrations. ATH, an Al(OH)_3_ modifier, is a standard flame retardant used in polymers. ATH was detected in all FPU samples, but it was found at varying levels in this work. It is a primary crystalline phase in the PST, PT, and PTS samples, occurring over the range 71.8–81.6 wt.%. (Table 2). In the PU, the secondary phase acting as a retardant was Na_12_[Al_12_Si_12_O_48_]·18H_2_O, known as the N-A-S-H (NASH) phase, which can also enhance mechanical and adhesive properties with respect to substrates. This N-A-S-H phase is added at a quantity of 17.6–19.1 wt.% towards 100% of the crystalline phase in the cases of the PST, PT, and PTS adhesives (Figure 3, Table 4). Its adhesive, mechanical, and retardant properties were also used in the PS and PM samples, but in smaller quantities (7.7–9.2 wt.%). Carbonates, which work as flame retardants by releasing CO_2_ gases and diluting fire reactants, were added mainly to the PS material, in the form of calcite and dolomite, and in a total quantity of 84.5%. For the other prepared FPUs, the weight fractions were below 5.5 wt.%.% (Table 2). XRD measurement confirmed the existence of the BaSO_4_ phase in the projected PU. According to the standard literature, this phase was primarily used to enhance the material’s mechanical properties and thermal stability, as was noted in this paper’s introduction. For the PST and PTS adhesives, this additive was present at 0.8 and 7.5 wt.%, respectively, but it was the main crystalline phase in PM polyurethane, accounting for 75.6%. The influence of this phase on thermal stability and the degree of FPU decomposition will be discussed in the subsequent “thermal” section of this work. The last recognised filler added to the PTS and PM materials was TiO_2_, at levels below 2 wt.%, as an anti-ageing agent. There is almost no data on the N-A-S-H additive with respect to FPU adhesives in the literature, and there is no data showing such a complex crystalline composition.

The identified crystalline phases may significantly influence the thermal stability and functional behaviour of the investigated polyurethane systems. The high content of gibbsite (Al(OH)_3_) detected in the PST, PT, and PTS samples is particularly important, because aluminium trihydroxide is a well-known mineral flame-retardant filler that undergoes endothermic dehydration with water release and demonstrates formation of alumina-type residues during heating [17,51]. This mechanism may reduce the local temperature of the degrading polymer and dilute combustible degradation products. Simultaneously, the hydrated N-A-S-H phase may also contribute to the thermal response by releasing physically retained and structurally bound water at elevated temperatures, as reported for sodium aluminosilicate hydrate systems [12]. In the PS sample, the dominant presence of calcite and dolomite is relevant to high-temperature behaviour, because carbonate phases decompose at temperatures higher than the main polyurethane degradation range, producing CaO/MgO-containing inorganic residues and CO_2_. Literature data show that calcite decomposition proceeds mainly above approximately 675–800 °C, while dolomite decomposition is also strongly temperature- and atmosphere-dependent [67,68]. Therefore, these phases may contribute to the stability of the inorganic residue and to partial dilution of combustible volatile products during late-stage degradation. The high BaSO_4_ content observed in the PM system should be interpreted primarily as evidence of a thermally stable, chemically inert mineral filler rather than as direct evidence of the improved intrinsic stability of the polyurethane matrix. Barium sulphate can increase the mineral fraction of the composite, thereby reducing the relative mass loss during heating. It may also contribute to stiffness and dimensional stability through a filler-reinforcement effect [11].

The XRD results indicate that the types and relative amounts of crystalline fillers are important factors controlling the thermal response and functional properties of the investigated polyurethane materials. However, these effects should be interpreted as phase-composition-related contributions to the composite behaviour, rather than as isolated effects of individual crystalline phases on the polyurethane matrix.

### 3.3. Thermal-Stability Analysis

**Figure 4:**
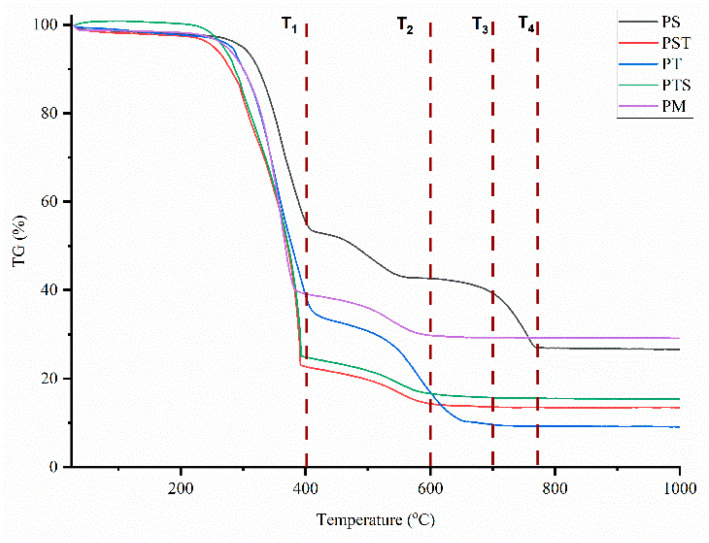
Polyurethane mass changes as a function of temperature, measured in air flow, and divided into decomposition steps.

**Figure 5:**
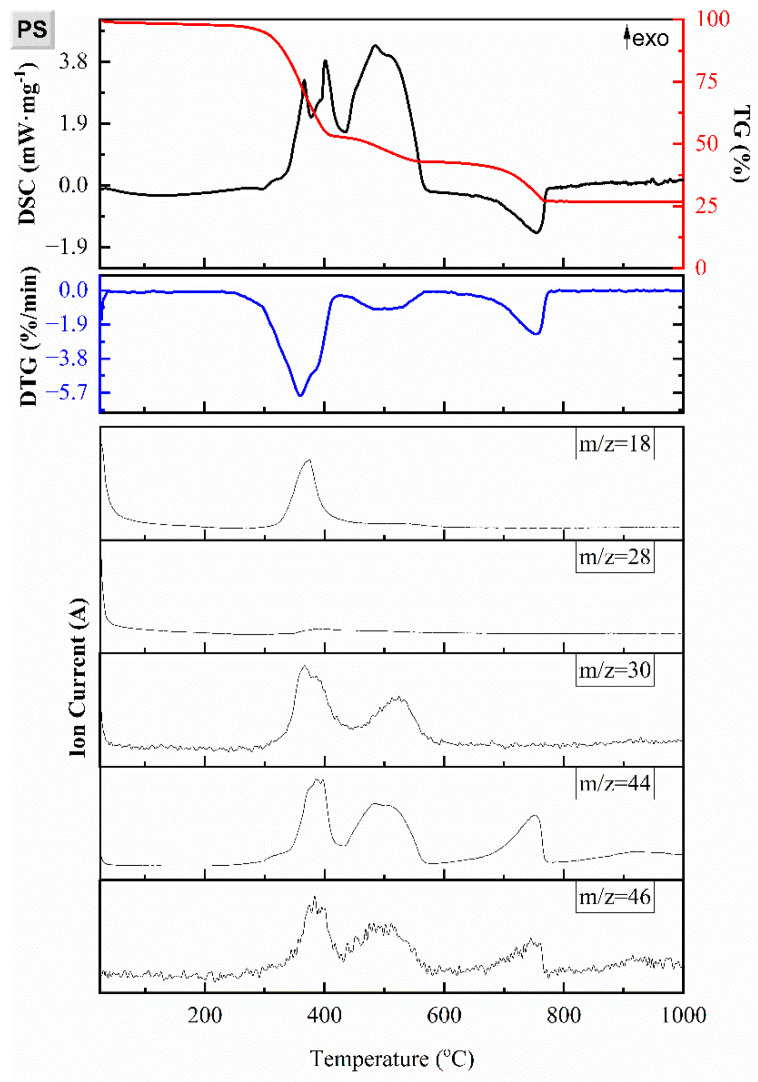
DSC-TG-QMS analysis of the PS material.

**Figure 6:**
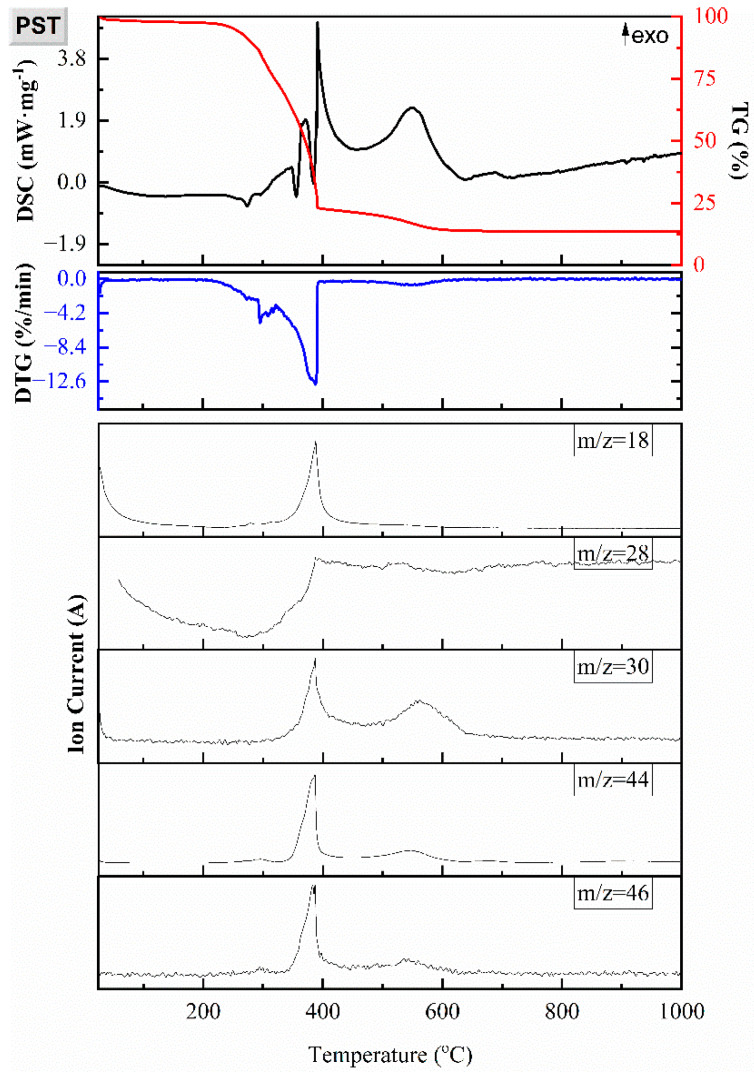
DSC-TG-QMS analysis of the PST material.

**Figure 7:**
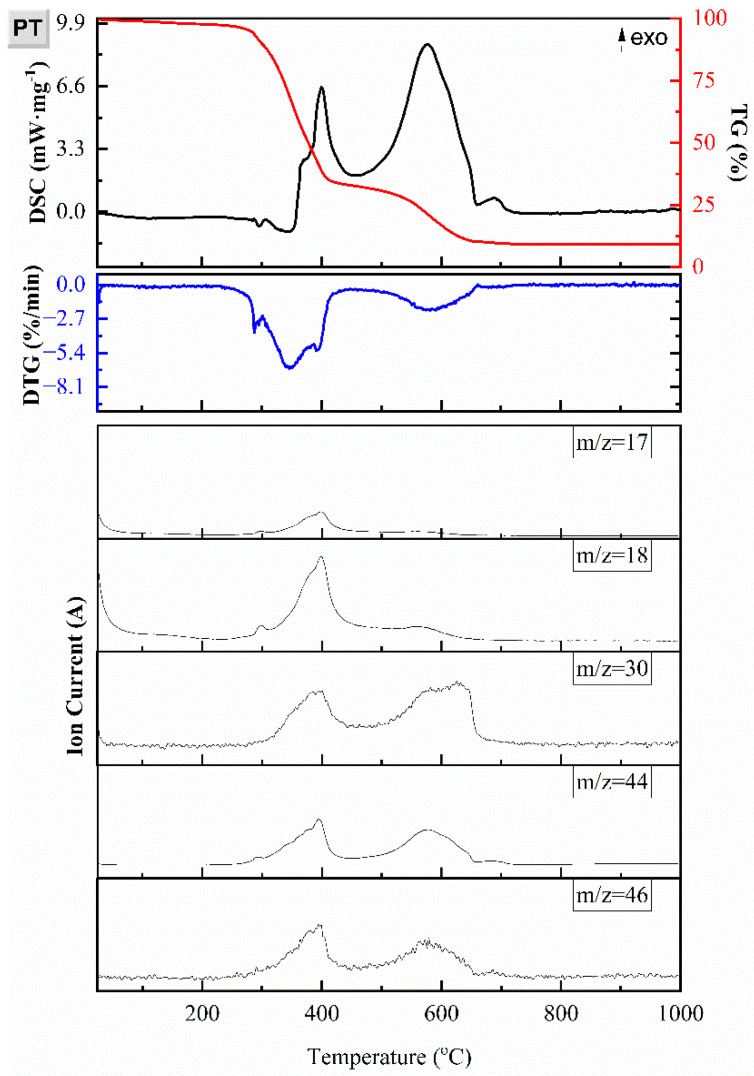
DSC-TG-QMS analysis of the PT material.

**Figure 8:**
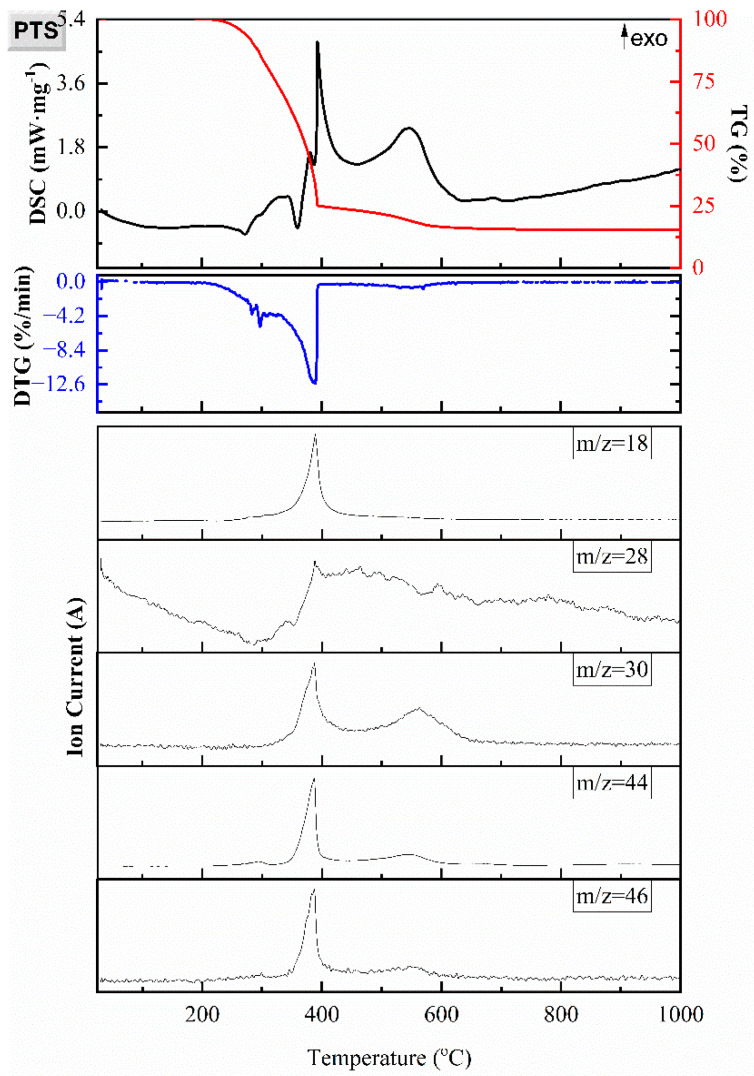
DSC-TG-QMS analysis of the PTS material.

**Figure 9:**
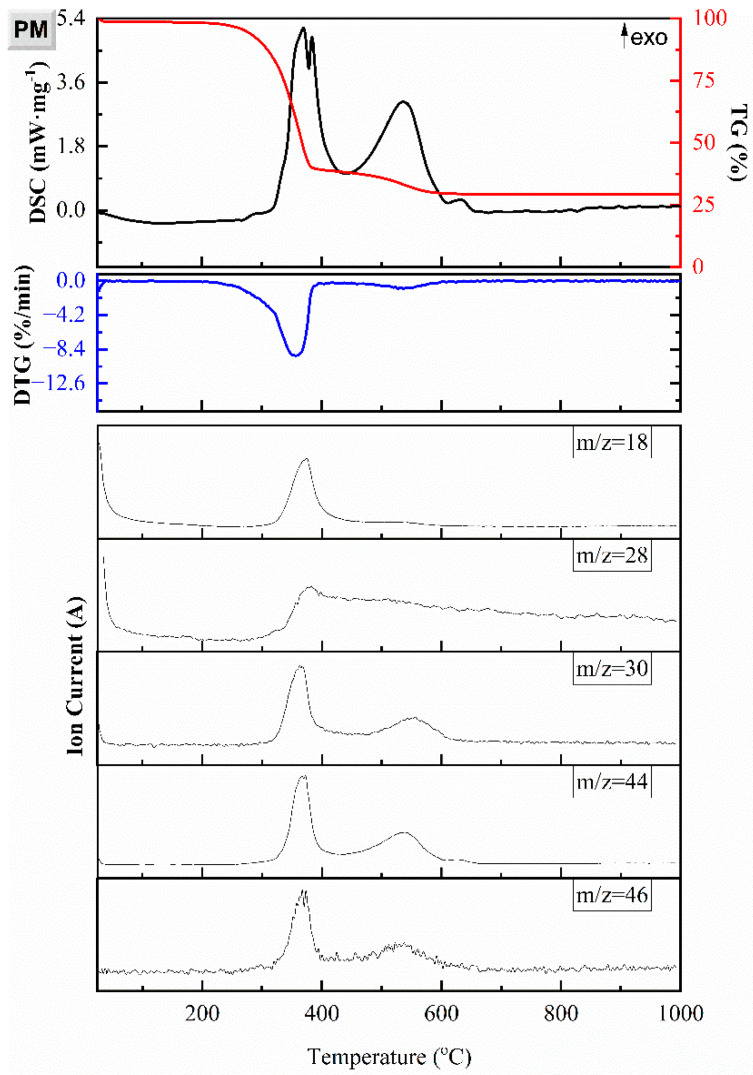
DSC-TG-QMS analysis of the PM material.

The thermal effects, mass changes, and related mass spectroscopy data are presented in Figure 4, Figure 5, Figure 6, Figure 7, Figure 8 and Figure 9. The mass change data, obtained from the TG curve shown in Figure 4, are presented in Table 5.

The thermal degradation of the investigated polyurethane systems proceeds through several overlapping stages associated with the decomposition of the organic polyurethane matrix and the thermal response of the inorganic filler phases. The first degradation stage, observed mainly up to approximately 300–350 °C, can be assigned primarily to the dissociation of thermally labile urethane linkages and the initial decomposition of hard-segment domains, accompanied by the release of low-molecular-weight products. In the investigated systems, this stage also overlaps with dehydration of hydrated mineral phases, particularly gibbsite and N-A-S-H, which release water during heating [12,17,51]. The next degradation stage, occurring mainly between approximately 350 and 500 °C, involves further decomposition of the polyurethane’s hard and soft segments, including isocyanate-derived structures and polyol-containing domains. This interpretation is consistent with the QMS signals assigned to CO_2_, CO, NO_x_, HCN, and CN-containing fragments, which indicate progressive scission and oxidation of urethane, isocyanate-derived, and polyol-related structures [17,18,69]. At temperatures above approximately 600 °C, the remaining mass changes are increasingly governed by the transformations of mineral fillers rather than by the polyurethane matrix itself. In carbonate-containing systems, especially PS, calcite and dolomite decomposition contribute to additional CO_2_ evolution and the formation of CaO/MgO-containing inorganic residues [67,68]. In contrast, BaSO_4_-rich compositions, especially PM, contain a thermally stable mineral phase that mainly increases the inorganic residue fraction and reduces the relative mass loss of the composite system, rather than directly improving the intrinsic chemical stability of the polyurethane matrix [11]. Thus, the degradation behaviour of the investigated materials should be interpreted as the combined effect of polyurethane network decomposition, evolution of gaseous degradation products, dehydration of hydrated fillers, and high-temperature transformations of carbonate or sulphate mineral phases.

Our last paper presented data with a heating rate of 5 °C· min^−1^, and the measurement was completed at 600 °C due to the use of aluminium crucibles. The measurement showed a two-step degradation in most cases [16]. FPU and filler components of the PU-based materials cause this behaviour. Due to the curved behaviour of, for example, PT polyurethane, it was decided to increase the temperature measurement range to 1000 °C, using the standard heating rate of 10 °C·min^−1^, with mass spectroscopy analysis. Looking at the TG curves (Figure 4), it is visible that the filler has a significant influence on the thermal behaviour of F&R PU. The curves show only three stages of material degradation in one case. In the analysis, the TG curves were divided into T_1_–T_4_ limiting temperatures that depended on the FPU type. Two materials, PS and PT, show mass change above 600 °C. In the case of the PS material, the mass change is stable only above 750 °C, and step T_2_–T_4_ is connected with the dolomite and calcite filler, which decompose sequentially at 675–800 °C and 600–800 °C (dolomite) at 10 °C·min^−1^ [67,68]. The decomposition of carbonates depends on the particle-size distribution and the heating rate; with a faster temperature rise, decomposition can extend to 950 °C, as with dolomite [68]. So, for the PS material, the final stage of FPU degradation is confirmed by XRD phase composition measurements (Figure 2 and Figure 3).

Also, gibbsite is used in varying amounts across all FPU types, and its decomposition depends on particle-size distribution and heating rate. At a high heating rate of 20 °C·min^−1^, decomposition starts at 250 °C, and this shifts to around 230 °C at slower heating rates [51]. So, the release of water vapour and the formation of alumina are indicated in the first stage of PU decomposition, which is especially evident for gibbsite, with higher concentrations in the PST, PT, and PT samples (Figure 4). An important role is also played by the N-A-S-H phase, which supplies water to the system. Similarly, evolved gases, as decomposition products, dilute reactive oxygen gases originating from FPU, thereby decreasing flame temperature. N-A-S-H can be similar to the N-C-A-S-H phase, and the first decomposition occurs up to 150 °C, during which free water evaporates from the gel. Bound water is removed at higher temperatures of around 260 °C [13]. By analysing the curves in Figure 4, it was found that the PT material shows a decomposition endpoint at a temperature 60 °C higher than those of PM, PTS, and PST. This indicates that small amounts of carbonate or sulphate filler can alter the final decomposition temperature of PM, PTS, and PST polyurethanes. This sulphate filler is probably responsible for lower polymer flexibility. The lower mass change in the PM sample is due to the barium sulphate additive, and its low-concentration effect is confirmed by the slight mass difference between the PST and PTS samples and by XRD measurements of the initial crystalline phase composition (Figure 4). Finally, any oxide products of filler decomposition can serve as fire retardants and enhance mechanical properties. It is worth noting that retardants that produce OH and H_2_O as products can replace carbonate fillers, which release CO_2_ during decomposition and can harm the environment. Also, the mass change during thermal treatment of FPUs depends on the structural decomposition of the polymeric component. That is why mass spectroscopy measurements were also performed on all samples. Polyurethanes generally begin to break down at temperatures between 200 °C and 300 °C. The first stage of decomposition involves the disintegration of the softer components, such as polyol segments. The next stage, which occurs between 350 °C and 450 °C, is characterised by the breakdown of the more rigid components, including urethane bonds. Once decomposition is complete, typically at temperatures above 600 °C, any remaining residues are usually attributable to fillers or additives. Moreover, the threshold temperature for the onset of thermal degradation in polyurethanes ranges from 220 °C to 270 °C. It is influenced by factors such as the type of polyol used and the degree of cross-linking. The mass-loss rate is influenced by the rate of temperature rise and the material’s specific formulation, with the peak mass loss typically observed between 300 °C and 450 °C, coinciding with degradation of both the hard and soft components. In the PS sample example, dolomite and calcium carbonate, as shown in the DSC-TG-QMS analysis curve, decompose at slightly above 600 °C, with CO_2_ release evident in the QMS spectra (Figure 5). The endothermic DSC peak also confirms the decomposition of carbonates. The QMS analysis also confirms that the water vapour originates from the gibbsite and N-A-S-H phases. The spectra of *m*/*z* = 30 and 46 are from NO_x_, where NO_2_ is formed and detected in small quantities. Small amounts of NO_2_ are confirmed by the instability (signal roughness) at the apparatus’s detection limit. The NO_2_ is also visible in the same temperature range as CO_2_, between 600 and 780 °C. There is no NO (*m*/*z* = 30) in this range—Figure 5. The isocyanate decomposition products are clearly visible in the ion current signal at *m*/*z* = 30 and are not present in the signal shown in Figure 5 at *m*/*z* = 26–27 [17,18].

In comparison to the PS sample, in the case of PST material (Figure 6), the CO_2_ QMS (*m*/*z* = 44) signal decreases above 600 °C, but the CO (*m*/*z* = 28) coming from PU is stable after above 300 °C. This is a confirmation of the lack of carbonate in the material composition. There are also peaks in the NO_x_ ion current (*m*/*z* = 30 and 46). The NO signal is much stronger than NO_2_. This signal appears at the boundary between the first and second FPU degradation steps and can be attributed to isocyanate groups, which confer rigidity to FPU. As mentioned in the introduction to this paper, isocyanate groups decompose during the second stage of FPU degradation. In isocyanates, there are functional groups, namely, N=C=O, which, during decomposition, cause the production of CO and NO_x_ gases. The OH^−^ (*m*/*z* = 17) ions are also visible in the place of water (*m*/*z* = 18). This situation can be explained by the thermal degradation of isolated HS (hard segments) into isocyanate, alcohols, primary and secondary amines, and olefins [17]. During degradation, CO_2_ is produced, as confirmed by QMS by a sharp peak at the same position as NO_X_ and water (Figure 6). The same paper confirms our QMS-recorded signal: the second step, in this case for the PST sample, also involves decomposing soft segments (SS). According to the cited paper, this decomposition of the urethane segment begins in the 150–200 °C range with the formation of primary and secondary alcohols. Thermal analysis is difficult to measure directly, but it can be inferred from the material’s shape using the HSM method, which is described later in this paper. Chattopadhyay and Webster [17] confirmed that this process is clearly evident in the TG curve above 200 °C. This process is finished below 300 °C. The polyol decomposition was confirmed above 400 °C. The HCN (*m*/*z* = 27) and CN (*m*/*z* = 26) signals in the temperature range 350–500 °C were also recorded.

A slightly different situation arises with the PT sample (Figure 7), which is a more rigid material. There is also a visible signal from OH^−^ and H_2_O gases, which arises from PU decomposition reactions in the PST sample and from N-A-S-H and gibbsite fillers. In accordance with Figure 3, this PT polyurethane contains a calcium carbonate additive that can influence the second-stage FPU decomposition and the endset temperatures of CO_2_ and NO_x_ gases, which are around 650 °C. In this FPU case, the second exothermic peak is stronger than in the PST material case. This is probably connected to the ratio of hard to soft segments. For more rigid materials, more groups are connected to hard segments [17]. In the case of such a connected structure of hard segments, the endset temperature of OH^−^ and H_2_O release is actually 550 °C. In that situation, the endset of the second step of NO_x_ release shifts to a higher temperature and is similar in height and shape to that of the first step (Figure 7). Different endsets and ratios for the second-to-first NO_x_ generation step are shown in Figure 5, Figure 6, Figure 8, and Figure 9 for the PS, PST, PTS, and PM materials, respectively. Generated HCN oxidises to carbon dioxide, water, NO, and N_2_O [69]. This process occurs over the range 507–1047 °C, but in the present case, it finishes below 700 °C for all PUs. Looking at TG, especially around 400 °C, it is interesting that the mass loss is less pronounced than in the more flexible PST (Figure 6) and PTS (Figure 8). Also, the DTG curve for the PT sample is much shallower and broader (Figure 7). At this temperature, for PST and PTS, the mass loss is sudden, and the QMS peaks for CO_2_ (*m*/*z* = 44) and NO_x_ (*m*/*z* = 30 and 46) are sharp. The last small peak, (Figure 7) for CO_2_ (*m*/*z* = 44) and NO_2_ (*m*/*z* = 46), can be linked to the decomposition of isocyanates, polyols, and residual organic phases, and to their reaction with oxygen, as evidenced by a small exothermic peak around 700 °C. The exact minimal exothermic peak on the DSC curve is also visible in the case of the PST material (Figure 6).

During the DSC-TG-QMS analysis (Figure 8), the PTS sample’s behaviour was very similar to that of the PST material (Figure 6). These samples, as shown by XRD analysis (Figure 3), differ in the amounts of N-A-S-H and gibbsite, and in the presence of BaSO_4_ and calcium carbonate, which are present only in the PTS sample (and not found in the PST sample). In the case of calcium carbonate (1.5 wt.%), there is no effect on the thermal analysis result, but it can have a slight negative effect on the thermal stability of barium sulphate. It is plausible that CaCO_3_ will influence lower mass loss in the first and second stages of FPU degradation, as in the case of the PS sample (Figure 5).

**Figure 10:**
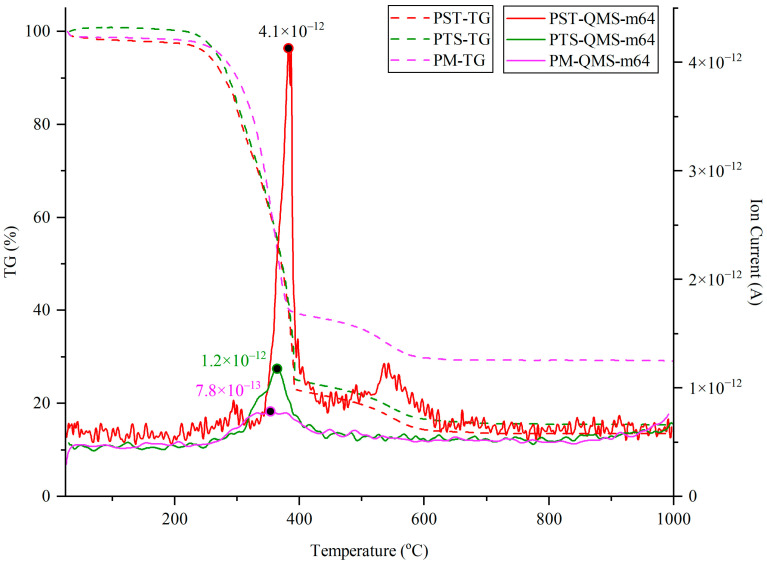
Analysis comparison of TG and SO_2_ ion current for the PST, PTS and PM materials containing barium sulphate in their filler compositions.

For the PM adhesive, the thermogravimetric derivative (Figure 9) indicates that decomposition is complete at 650, at which point the final quantities of gaseous products are released. The last small exothermic DSC peak around 625 °C is attributed to CO_2_ (*m*/*z* = 44) and is explained by the reaction of residual polymer decomposition products with oxygen after the graphite protective layer is burned above 400 °C. On the DSC curve, the first decomposition exothermic signal, around 300–400 °C, comprises two peaks. These peaks overlap on the double, slightly visible peak in the NO_2_ (*m*/*z* = 46) QMS ion current case—Figure 9. The QMS signals at *m*/*z* = 18, 28, 30, 44, and 46 arise from water, CO_x_, and NO_x_ gases released during the decomposition of the constituent phases of organic and inorganic materials. This is similar to the cases of other FPU materials described in this paper. So, the decomposition of gibbsite and the N-A-S-H phase will produce water vapour (*m*/*z* = 18). Some barium sulphate can react with this water, as evidenced by the QMS *m*/*z* = 64 signal shown in Figure 10 and described later. The change in reduced mass in this PM sample relative to PT, PST, and PTS is due to lower fire-retardant levels. Solid inorganic baryte filler is responsible for the material’s improved mechanical properties and enhanced gas-diffusion pathways at the onset of decomposition, as explained further in the HSM analysis.

The interesting signal from QMS analysis (Figure 10) shows differences in the decomposition signal for *m*/*z* = 64. Figure 10 compares *m*/*z* = 64 for PST, PTS, and PM samples containing various quantities of barium sulphate in the crystalline material. The table for BaSO_4_ shows that it is at 0.8 wt.% for PST, 7.5 wt. % for PTS and 75.6 wt.% for the PM material. In contrast, the maximum peaks of the recorded signals are inversely proportional to the sulphate contents. This can be linked to the lower amount of water extracted from the PM sample, where the combined amount of the N-A-S-H phase and gibbsite does not exceed 17%. In the PST sample, the PTS material exceeds 90%. So, in the PST sample, the signal at *m*/*z* = 64 from barium sulphate products in water is the strongest. The signals of gases connected with *m*/*z* = 64, which are harmful, are small in comparison to the OH^−^, H_2_O, CO and CO_2_ signals.

For comparison of the degradation behaviour of the investigated polyurethane systems, a comparative summary of the main DSC-TG-QMS observations and phase-related interpretation is additionally presented in Table 6.

**Figure 11:**
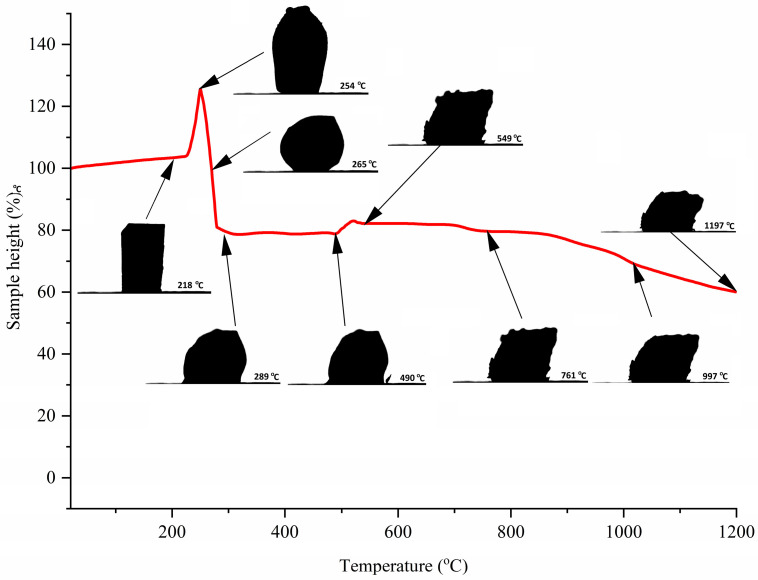
Combined presentation of the results obtained from two complementary thermal-analysis methods: dilatometric analysis and hot-stage microscopy (HSM). The red curve represents the relative linear dimensional changes of the PS polyurethane sample as a function of temperature obtained from dilatometric measurements, while the black images correspond to HSM micrographs recorded at the temperatures indicated by the arrows on the curve. The HSM images illustrate the temperature-dependent evolution of the sample shape during thermal treatment.

**Figure 12:**
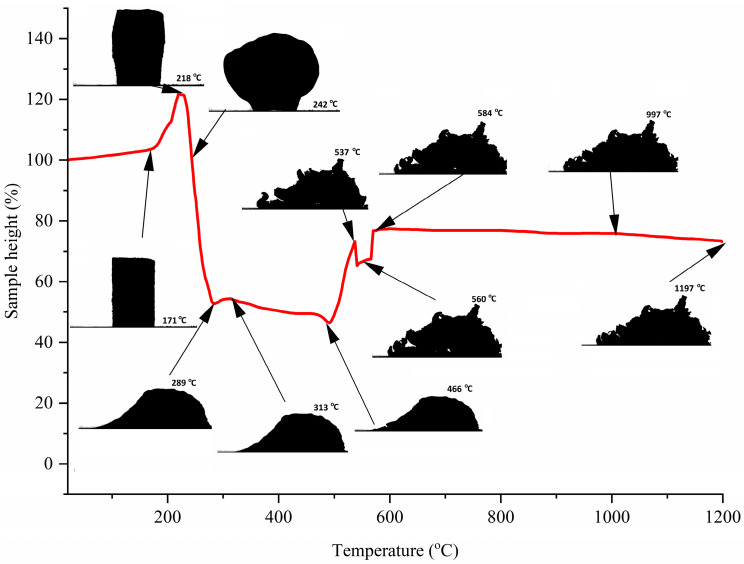
Combined presentation of the results obtained from two complementary thermal-analysis methods: dilatometric analysis and hot-stage microscopy (HSM). The red curve represents the relative linear dimensional changes of the PST polyurethane sample as a function of temperature obtained from dilatometric measurements, while the black images correspond to HSM micrographs recorded at the temperatures indicated by the arrows on the curve. The HSM images illustrate the temperature-dependent evolution of the sample shape during thermal treatment.

**Figure 13:**
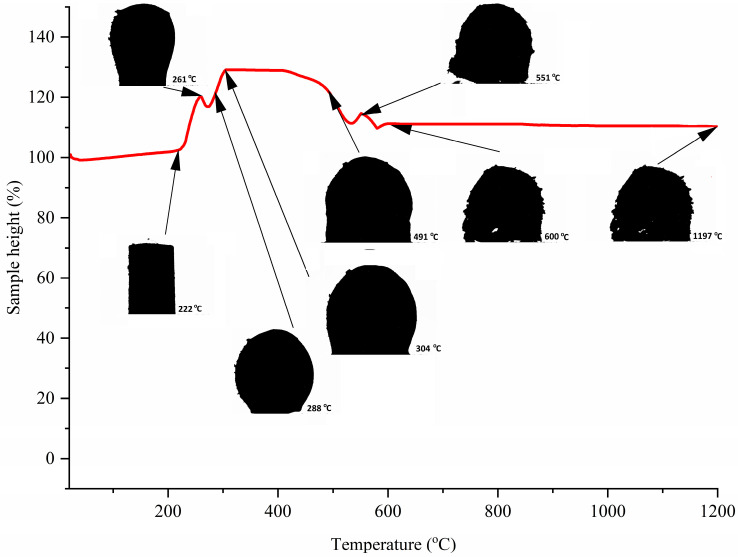
Combined presentation of the results obtained from two complementary thermal-analysis methods: dilatometric analysis and hot-stage microscopy (HSM). The red curve represents the relative linear dimensional changes of the PT polyurethane sample as a function of temperature obtained from dilatometric measurements, while the black images correspond to HSM micrographs recorded at the temperatures indicated by the arrows on the curve. The HSM images illustrate the temperature-dependent evolution of the sample shape during thermal treatment.

**Figure 14:**
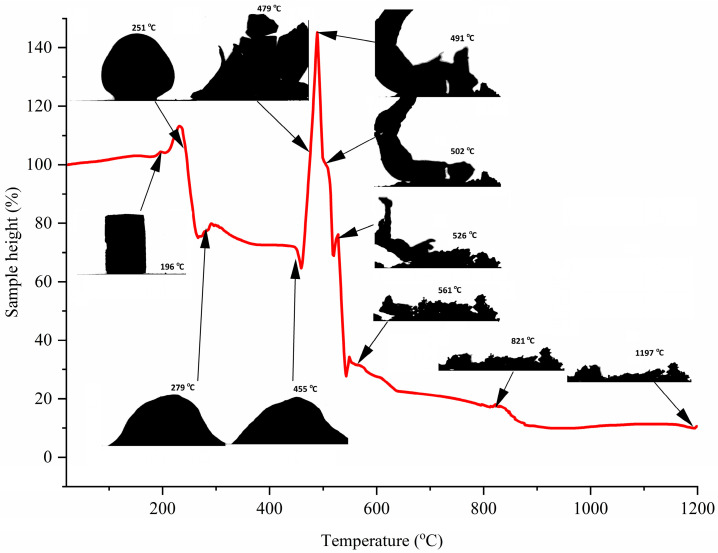
Combined presentation of the results obtained from two complementary thermal-analysis methods: dilatometric analysis and hot-stage microscopy (HSM). The red curve represents the relative linear dimensional changes of the PTS polyurethane sample as a function of temperature obtained from dilatometric measurements, while the black images correspond to HSM micrographs recorded at the temperatures indicated by the arrows on the curve. The HSM images illustrate the temperature-dependent evolution of the sample shape during thermal treatment.

**Figure 15:**
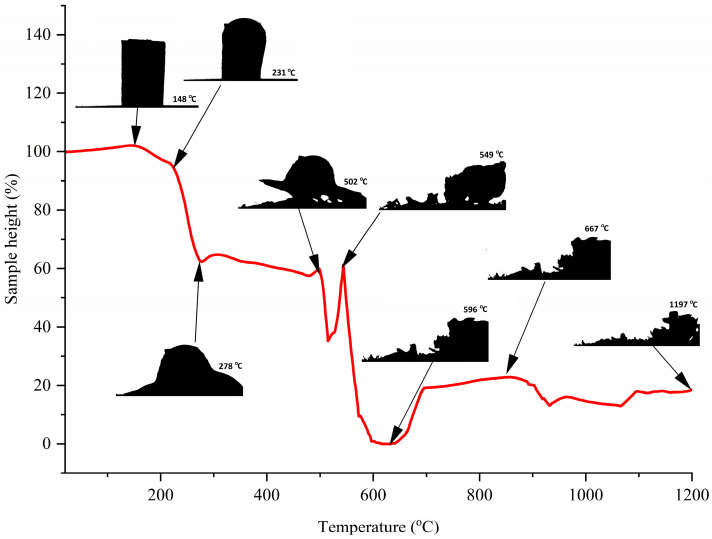
Combined presentation of the results obtained from two complementary thermal-analysis methods: dilatometric analysis and hot-stage microscopy (HSM). The red curve represents the relative linear dimensional changes of the PM polyurethane sample as a function of temperature obtained from dilatometric measurements, while the black images correspond to HSM micrographs recorded at the temperatures indicated by the arrows on the curve. The HSM images illustrate the temperature-dependent evolution of the sample shape during thermal treatment.

As mentioned in the paper, structural and thermal analyses, along with evolved-gas measurements, provide fundamental information on the material’s shape and thermal stability. That is why the polyurethane samples were representative of poured FPU material and were subjected to shape–thermal-stability analysis using hot-stage microscopy. The recorded linear changes and sample visualisation are illustrated in the HSM measurements shown in Figure 11, Figure 12, Figure 13, Figure 14 and Figure 15.

In the case of the PS sample, the material heated at 10 °C·min^−1^ remained stable up to 230 °C during HSM measurements, with a stable expansion rate. Above this temperature (Figure 11), the PS sample expands and swells rapidly, reaching a maximum at 250 °C, with no mass loss. TG analysis confirmed that the thermal-stability temperature reached 250 °C (Figure 4). In our earlier measurements, at a two-fold-lower heating rate, the thermal stability was 200 °C, which can be explained by the analysed material’s heat capacity and low thermal conductivity/diffusivity [16]. So, even if the TG curve does not show mass loss, the material becomes shape-unstable due to decreased rigidity from bond breaking and a lack of accessible water-vapour transport paths in the N-A-S-H phase. Further loosening of the PU structure makes diffusion patterns for water release from gibbsite and N-A-S-H decomposition [13,51]. The sample becomes round and, at higher temperatures up to 400 °C, exhibits high substrate wetting. The temperature of 250 °C is also indicated by a sharp mass loss on the TG curve up to 410 °C (Figure 4 and Figure 5). At this temperature, PU decomposes to isocyanate, amin and HCN (Figure 5), which is visible by a strong QMS-measured NO_x_ signal at *m*/*z* = 30. The NO_x_ releases samples up to 600 °C; as mentioned before, NO_2_ was recorded up to 800 °C but in meagre quantities (Figure 5). Any oxide products resulting from the degradation of material components will act as a filler, maintaining the mechanical properties of PU and improving gas diffusion pathways and the material’s thermal stability. Between 350 and 500 °C, a plateau in measured sample height can be associated with the continued presence of calcium carbonate and oxide products of file decomposition, improving the sample’s mechanical shape stability and creating gas diffusion paths. The slight decrease in material height above 600 °C, as measured by HSM (Figure 11), is attributed to the decomposition of dolomite and calcium carbonate, as confirmed by works in the literature [67,68] and our QMS analysis (Figure 5). There is also a slight decrease in sample height at temperatures above 800 °C, attributable to residual oxidation of PU.

As recorded by XRD analysis, the PST sample contains higher amounts of gibbsite and N-A-S-H rather than carbonates. It results in reduced material shape stability at low temperatures due to the absence of inorganic fillers that remain stable at such temperatures, such as calcium carbonate. The substrate wetting angle of molten PU is lower in the temperature range of 250–500 °C, and the closed material surface is stable due to low material mass loss in this temperature range (Figure 5 and Figure 12). At about 500 °C, there is a jump in material height (Figure 12) due to gas release and further degradation of decomposed PU, where the pressure of closed gaseous products causes material destruction. Compared with the PS material, there are no significant changes in sample height or shape above 600 °C, due to the absence of dolomite and calcium carbonate in the material’s initial composition (Figure 3, Figure 5 and Figure 6). No gases are released above this temperature (Figure 6 and Figure 12).

Different behaviour during HSM measurement was shown by the PT adhesive. The PT material, the thermal behaviour of which is shown in Figure 13, is the most rigid among the prepared PU samples. The HSM analysis shows that the material does not show a decrease in height. This material consists of polymer parts with only adhesion and fire-protection phases, such as gibbsite, N-A-S-H, and calcium carbonate. This material linearly expands up to 210 °C. Above this temperature, the material’s linear dimensions increase sharply up to 300 °C, then plateau until 400 °C. The increase in material size and swelling is associated with gibbsite decomposition, the glass transition, and bond breaking, leading to softening of the material and a lack of diffusion paths for evolved water vapour. Above 400 °C, there is a decrease in the material’s linear dimension, probably due to evaporating water diffusion and PU decomposition (Figure 4 and Figure 7). Figure 13 shows that sample roughness increases with temperature, which can be attributed to further PU decomposition, burning of the protective carbon layer, aluminium oxide formation (at low temperatures), and slow decomposition of the low amount of calcium carbonate (2.1 wt.%). Oxide formation from gibbsite decomposition improves mechanical properties and gas diffusion paths.

During hot-stage microscopy examinations, the same problem was encountered with PTS and PM samples at higher temperatures. The material height change and behaviour visualisation (recorded images) are shown in Figure 14 and Figure 15. The PTS material’s stable and controlled thermal expansion is maintained up to 200 °C (Figure 14), and in the case of the PM polyurethane sample, up to 150 °C (Figure 15). These samples have similar TGA curve shapes (Figure 4), but PM polyurethane shows the lowest shape stability compared to PS, PST, PT, or PTS. The PM sample also shows a smaller mass change than the PST and PTS samples. PTS and PM samples, as shown by TGA curves, exhibit a sudden, profound, and similar mass loss from 230 to 395 °C. These samples, containing five types of fillers, exhibit similar thermogravimetric behaviour but differ significantly in composition. In the case of PTS, the composition is mainly based on the flame retardant Al(OH)_3_ at 70.8 wt.%. Still, in the case of PM, there is mechanically responsible barium sulphate in 75.6 wt.% quantity. These phases are responsible for the first stage of material shape instability.

The HSM investigated PST material, the behaviour of which, presented in Figure 14, shows a slight increase in material height between 210 and 230 °C related to structure tightness, and high flexibility. Closing in the evaporating free water causes the material to swell, as visible in the microscopy images in Figure 14. Above 230 °C, large amounts of gibbsite and the N-A-S-H phase are decomposed [13]. This stage finishes at around 270 °C. Above this temperature, the second decomposition stage (Figure 4) occurs, during which the material’s shape changes slightly, up to 450 °C (Figure 14). When the temperature exceeds 450 °C, the material suddenly disintegrates. Further material changes are captured by FPU decomposition; above 800 °C, there is a slight decrease in material height. This change above 800 °C is caused only by the decomposition of a small amount of calcite. Barium sulphate most likely remains stable until the end of the measurement—its amount decreases as the height of the material towards the PST sample changes (Figure 4 and Figure 12).

In the case of PM adhesive (Figure 15), a large amount of barium sulphate causes a weakening of the structure, which at elevated temperatures results in the formation of diffusion pathways for the gas, especially water in the first stage, water coming from the N-A-S-H phase at 150 °C [13] and water coming from the decomposition of aluminium hydroxide at 230 °C [51]. So, the material does not suddenly swell like in the case of the other FPU—in this case, diffusion paths, provided by a large amount of barium sulphate, result in material shrinkage just below 200 °C (Figure 15). The first drop in sample height, of about 40%, occurs at approximately 270 °C, coinciding with the second stage of N-A-S-H phase decomposition and the release of bound water [13]. The microscopic image of the sample recorded at this temperature indicates a loss of shape and a decrease in the contact angle of the substrate on which the measured material lies. Further, the change in material height is slight up to 500 °C. Only above this temperature can FPU degradation significantly contribute to shape instability, as evidenced by the sudden change in shape of the tested material. Above 850 °C, a slight decrease is attributed to the decomposition of small amounts of CaCO_3_ and to the reduced thermal stability of BaSO_4_ in the reductive atmosphere induced by carbonate [10].

The HSM observations indicate that the investigated polyurethane systems differ not only in their thermal decomposition temperatures but also in the mechanisms of thermally induced structural destabilisation. Materials containing carbonate phases, particularly PS and, partially, PT, exhibited slower high-temperature dimensional changes and retained their macroscopic shape stability over a broader temperature range. In contrast, polyurethane systems rich in hydrated phases such as gibbsite and N-A-S-H exhibited more pronounced swelling and rapid structural destabilisation during the intermediate stages of degradation, which can be associated with the intensive release of gaseous decomposition products and water vapour. The PM system, with a high BaSO_4_ fraction, exhibited a different degradation pathway, which was characterised by earlier dimensional shrinkage and reduced swelling intensity. This behaviour may be related to the presence of a thermally stable mineral framework, which may affect the release and transport of gaseous decomposition products. These observations suggest that the crystalline phase composition influences not only the thermal stability itself but also the mode of thermally induced structural transformation in polyurethane systems during heating.

## 4. Conclusions

Studies have shown that fillers can significantly influence the decomposition behaviour, thermal stability, and dimensional response of polyurethane adhesives. Different applications of polyurethane adhesives require materials with varying stiffness or elasticity and, therefore, different flexibility-to-rigidity ratios, which directly affect the physicochemical properties of the systems under investigation. The comparison of structural, phase, and thermal analyses demonstrated that the combined use of FTIR, XRD, DSC-TG-QMS, and HSM constitutes a valuable, complementary methodological approach for characterising polyurethane adhesives intended for wooden structural applications.

The semi-quantitative FTIR analysis of the carbonyl stretching region revealed clear differences in hydrogen-bond organisation between the investigated polyurethane systems. The PT sample exhibited the highest fraction of associated carbonyl groups (F_assoc_ = 0.751) and the highest hydrogen-bonding index (HBI = 3.018), indicating the strongest contribution from hydrogen-bonded hard-segment environments. In contrast, PM showed the lowest carbonyl association (F_assoc_ = 0.432; HBI = 0.760). These structural differences were reflected in the thermal degradation behaviour and dimensional stability observed during heating. XRD analysis confirmed substantial differences in the crystalline phase composition of the investigated materials. PST, PT, and PTS systems were dominated by gibbsite (70.8–81.6 wt.% of the crystalline fraction) and N-A-S-H phases (17.6–19.1 wt.%), while PS contained high amounts of calcite and dolomite (57.5 and 27 wt.%, respectively). The PM sample was characterised by the highest BaSO_4_ content (75.6 wt.%). The obtained results demonstrated that the type and quantity of mineral fillers strongly affected the degradation pathways and thermal response of the polyurethane systems. In particular, the carbonate-rich PS material exhibited an additional high-temperature mass-loss stage between 660 and 770 °C (−14.4%), which was attributed to the decomposition of calcite and dolomite. In contrast, PST and PTS exhibited only minor mass changes above 600 °C, whereas PM showed the lowest relative mass loss, due to the presence of the thermally stable BaSO_4_ filler. The thermal analyses demonstrated that the main degradation of polyurethane systems occurred predominantly below approximately 410 °C, whereas the subsequent high-temperature behaviour depended mainly on the mineral phase composition. QMS measurements confirmed the release of H_2_O, CO_2_, CO, and NO_x_ during degradation, while HCN/CN-related fragments were additionally detected in selected systems, particularly PST. The results also showed that hydrated fillers, such as gibbsite and N-A-S-H, significantly affected water release during heating and influenced swelling and thermally induced destabilisation. The HSM investigations revealed clear differences in dimensional stability among the polyurethane systems. PS and PT retained macroscopic shape stability over a broader temperature range. In contrast, PST and PTS exhibited pronounced swelling and faster structural destabilisation, accompanied by intense gas evolution from hydrated phases. In contrast, PM exhibited earlier shrinkage and lower swelling intensity, which may be attributable to a thermally stable BaSO_4_-rich mineral framework that affects gas transport during decomposition.

The results confirmed clear relationships among polyurethane structure, crystalline phase composition, evolved-gas generation, and thermal shape stability. The study additionally demonstrated that hydrated mineral fillers that release water during decomposition may partially reduce the need for carbonate-based fillers, which are associated with CO_2_ evolution. However, further environmental and long-term durability studies are still required.

## Figures and Tables

**Table 1:** The characteristics of the bands in the examined polyurethane groups.

Wave Number (cm^−1^)	Band Group	Description
800–900	Backbone structure	C-H bending vibrations related to methyl or hydrocarbon groups
1100–1200	Backbone structure	C-O-C stretching vibrations, related to ether groups in polyol segments
1200–1300	Chemical groups	C-N stretching vibrations and vibrations related to C-O stretching of urethane groups
1450–1500	Deformations and structure	CH_2_ bending vibrations and vibrations related to structural deformation
1600–1650	Hard and soft segments	Aromatic groups building FPU structure
1700–1750	Chemical groups	Carbonyl (C=O) stretching vibrations assigned to urethane groups

**Table 2:** Deconvoluted band assignment for the C=O region.

Components	Bands, cm^−1^	Assignments
A	1642 + 1650 + 1668	Strongly associated/urea-related C=O
B	1703 + 1718	H-bonded urethane C=O envelope
C	1728 + 1739	Free/weakly H-bonded urethane C=O

**Table 3:** Semi-quantitative parameters calculated from the deconvoluted C=O stretching region.

Sample	F_strong_	FH-Bonded	F_assoc_ = F_strong_ + FH-Bonded	F_free_	HBI = F_assoc_/F_free_
PM	0.138	0.293	0.432	0.568	0.760
PS	0.083	0.492	0.575	0.425	1.354
PST	0.184	0.321	0.505	0.495	1.021
PT	0.122	0.629	0.751	0.249	3.018
PTS	0.258	0.309	0.568	0.432	1.313

**Table 4:** Phase composition analysis of polyurethanes, performed at RT.

Sample Name	Compound Name	Chemical Formula	Composition of Crystalline FPU Part wt%	ICDD Card
PS	Calcite	CaCO_3_	57.5	01-086-4274
	Dolomite	CaMg(CO_3_)_2_	27	00-036-0426
	Sodium Calcium Aluminium Silicon Oxide Hydrate	Na_12_[Al_12_Si_12_O_48_]·18H_2_O	9.0	04-018-9254
	Gibbsite	Al(OH)_3_	6.6	01-080-6432
PST	Gibbsite	Al(OH)_3_	81.6	01-080-6432
	Sodium Calcium Aluminium Silicon Oxide Hydrate	Na_12_[Al_12_Si_12_O_48_]·18H_2_O	17.6	04-018-9254
	Baryte	BaSO_4_	0.8	04-007-7651
PT	Gibbsite	Al(OH)_3_	79.7	01-080-6432
	Sodium Calcium Aluminium Silicon Oxide Hydrate	Na_12_[Al_12_Si_12_O_48_]·18H_2_O	18.2	04-018-9254
	Calcite	CaCO_3_	2.1	01-086-4274
PTS	Gibbsite	Al(OH)_3_	70.8	01-080-6432
	Sodium Calcium Aluminium Silicon Oxide Hydrate	Na_12_[Al_12_Si_12_O_48_]·18H_2_O	19.1	04-018-9254
	Baryte	BaSO_4_	7.5	04-007-7651
	Calcite	CaCO_3_	1.5	01-086-4274
	Rutile	TiO_2_	1.2	00-034-0180
PM	Baryte	BaSO_4_	75.6	04-007-7651
	Sodium Calcium Aluminium Silicon Oxide Hydrate	Na_12_[Al_12_Si_12_O_48_]·18H_2_O	9.2	04-018-9254
	Gibbsite	Al(OH)_3_	7.7	01-080-6432
	Calcite	CaCO_3_	5.5	01-086-4274
	Rutile	TiO_2_	2.0	00-034-0180

**Table 5:** Thermal degradation of FPU, divided into mass-loss steps. The thick black line marks the end of material decomposition.

Step	Temperature (°C)	Mass Loss (%)				
		PS	PST	PT	PTS	PM
RT	25	0	0	0	0	0
RT–T_1_	410	−46.5	−77.6	−64.6	−75.5	−61.1
T_1_–T_2_	600	−10.8	−8.1	−18.5	−8.0	−9.1
T_2_–T_3_	660	−1.2	−0.5	−6.7	−0.7	−0.5
T_3_–T_4_	770	−14.4	−0.3	−0.9	−0.3	0
T_4_–End	1000	−0.4	−0.1	−0.2	−0.2	−0.1

**Table 6:** Comparative summary of DSC-TG-QMS results and phase-related interpretation of the thermal behaviour of the investigated polyurethane systems.

Sample	TG Mass-Loss Profile Based on Table 5	Main Interpretation Consistent with DSC-TG-QMS Data	Main QMS Signals Discussed in the Manuscript	Crystalline Phases Identified by XRD
PS	−46.5% up to 410 °C; −10.8% between 410–600 °C; pronounced additional loss of −14.4% between 660–770 °C	Main FPU degradation occurs below 410 °C; the high-temperature mass loss is attributed mainly to carbonate decomposition	H_2_O, CO_2_, NO_x_, CO	Calcite, dolomite, N-A-S-H, gibbsite
PST	−77.6% up to 410 °C; −8.1% between 410–600 °C; negligible mass loss above 600 °C	Predominant FPU degradation below 410 °C, with contribution from dehydration of gibbsite/N-A-S-H; no evident carbonate-related high-temperature mass-loss step	H_2_O, CO_2_, CO, NO_x_, HCN/CN-related fragments; *m*/*z* = 64 discussed for BaSO_4_-containing samples	Gibbsite, N-A-S-H, trace BaSO_4_
PT	−64.6% up to 410 °C; substantial additional loss of −18.5% between 410–600 °C and −6.7% between 600–660 °C	Broader degradation behaviour compared with PST/PTS, consistent with a more rigid FPU system and delayed evolution of degradation products	H_2_O, CO_2_, NO_x_, CO	Gibbsite, N-A-S-H, minor calcite
PTS	−75.5% up to 410 °C; −8.0% between 410–600 °C; negligible mass loss above 600 °C	Thermal behaviour similar to PST; degradation dominated by FPU decomposition and hydrated filler dehydration, with minor contribution from BaSO_4_/calcite-containing filler system	H_2_O, CO_2_, CO, NO_x_; *m*/*z* = 64 discussed for BaSO_4_-containing samples	Gibbsite, N-A-S-H, BaSO_4_, minor calcite, TiO_2_
PM	−61.1% up to 410 °C; −9.1% between 410–600 °C; negligible mass loss above 660 °C	FPU degradation occurs mainly below 410 °C in a BaSO_4_-rich mineral system; high BaSO_4_ content contributes mainly to the inorganic residue fraction and reduced relative mass loss	H_2_O, CO_2_, CO, NO_x_; *m*/*z* = 64 discussed for BaSO_4_-containing samples	BaSO_4_, N-A-S-H, gibbsite, calcite, TiO_2_

## Data Availability

Data supporting the reported results, including data generated during the study, are available at https://doi.org/10.58032/AGH/FRFXP1.

## Supplementary Materials

The following supporting information can be downloaded at: https://www.mdpi.com/article/10.3390/polym18111396/s1, Figure S1: Deconvoluted spectrum of PM sample; Figure S2: Deconvoluted spectrum of PS sample; Figure S3: Deconvoluted spectrum of PST sample; Figure S4: Deconvoluted spectrum of PT sample; Figure S5: Deconvoluted spectrum of PTS sample.

## Abbreviations

The following abbreviations are used in this manuscript:

PU	Polyurethane
FPU	Flexible Polyurethanes
FTIR	Fourier Transform Infrared Spectroscopy
XRD	X-ray Diffraction
DSC	Differential Scanning Calorimetry
TGA	Thermogravimetric Analysis
QMS	Quadrupole Mass Spectrometry
HSM	Hot-Stage Microscopy
N-A-S-H	Sodium Aluminosilicate Hydrate
TG	Thermogravimetric curve
DTG	Derivative Thermogravimetry
