# Supplementary material for: Relation Between Thermal Analysis, Phase Composition and Structure of Polyurethane Adhesives for Application in Wooden Structural Joints
Source: Polymers (Basel). 2026 Jun 4;18(11):1396. doi: 10.3390/polym18111396 (PMC13258856; doi:10.3390/polym18111396)
Supplement: Supplementary file 1 [file polymers-18-01396-s001.zip › polymers-4307360-supplementary.pdf]

Supplementary Information

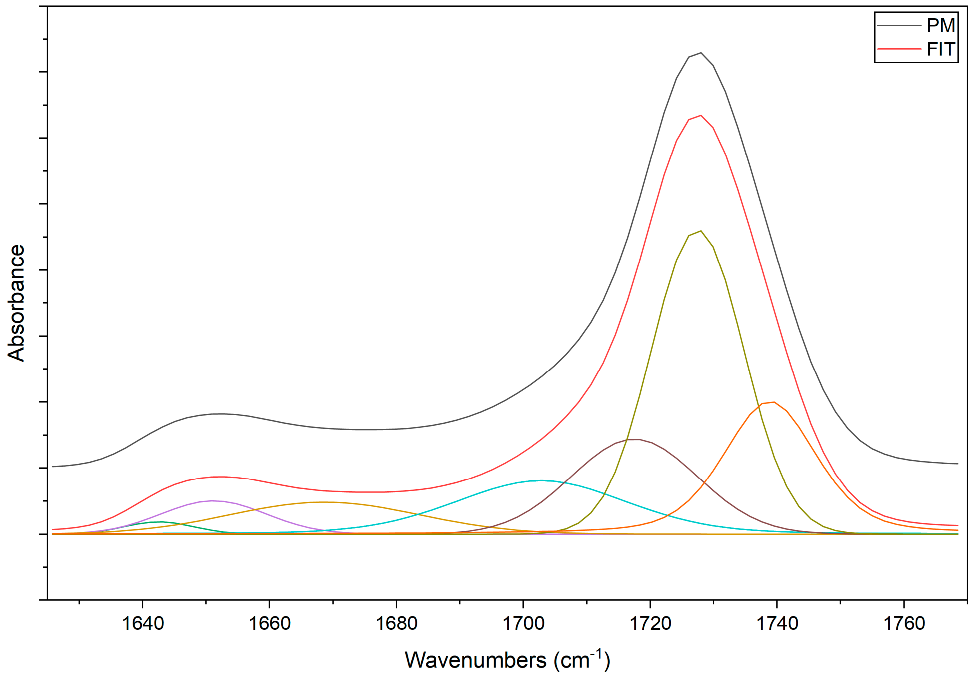

Figure S1. Deconvoluted spectrum of PM sample.

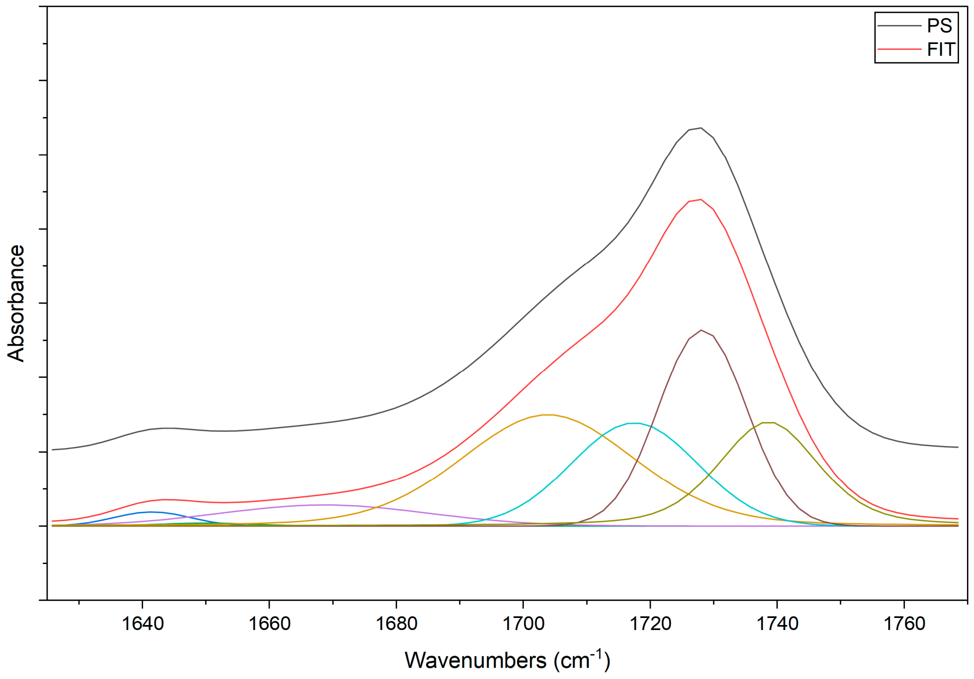

Figure S2. Deconvoluted spectrum of PS sample.

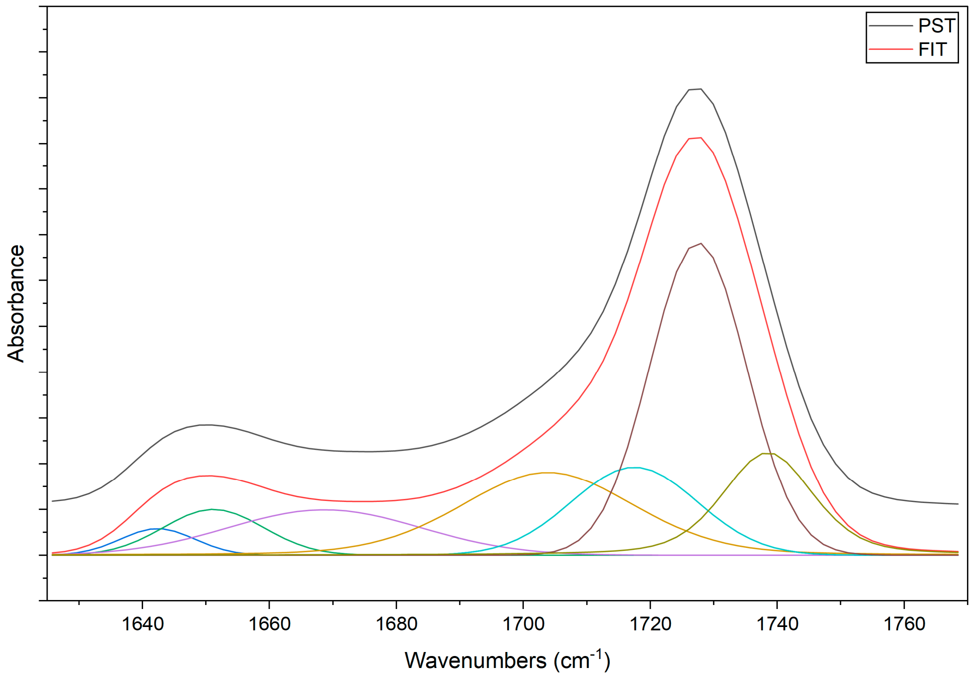

Figure S3. Deconvoluted spectrum of PST sample.

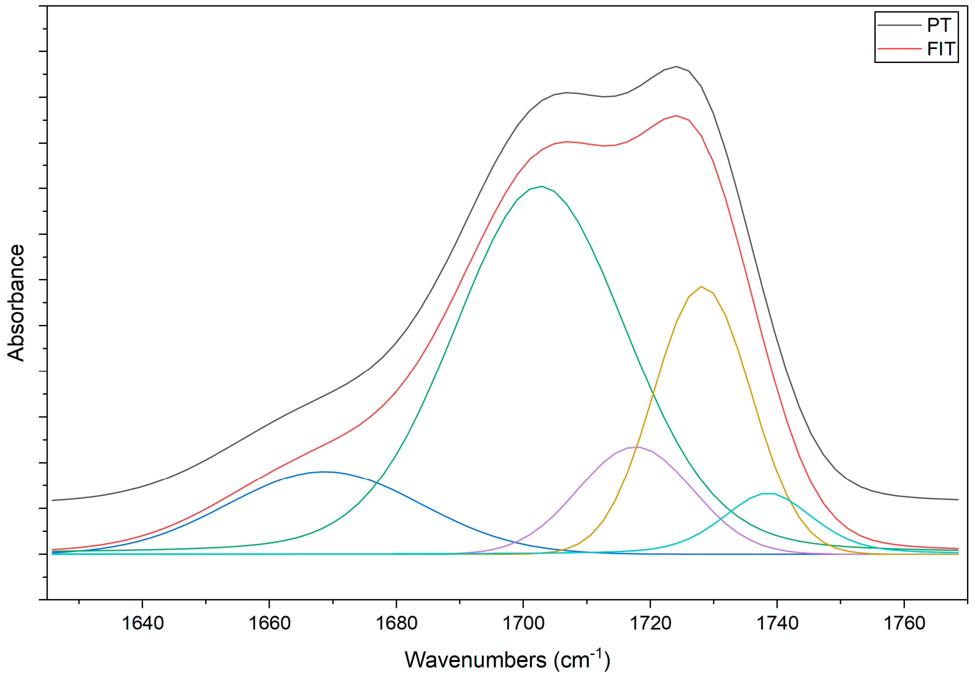

Figure S4. Deconvoluted spectrum of PT sample.

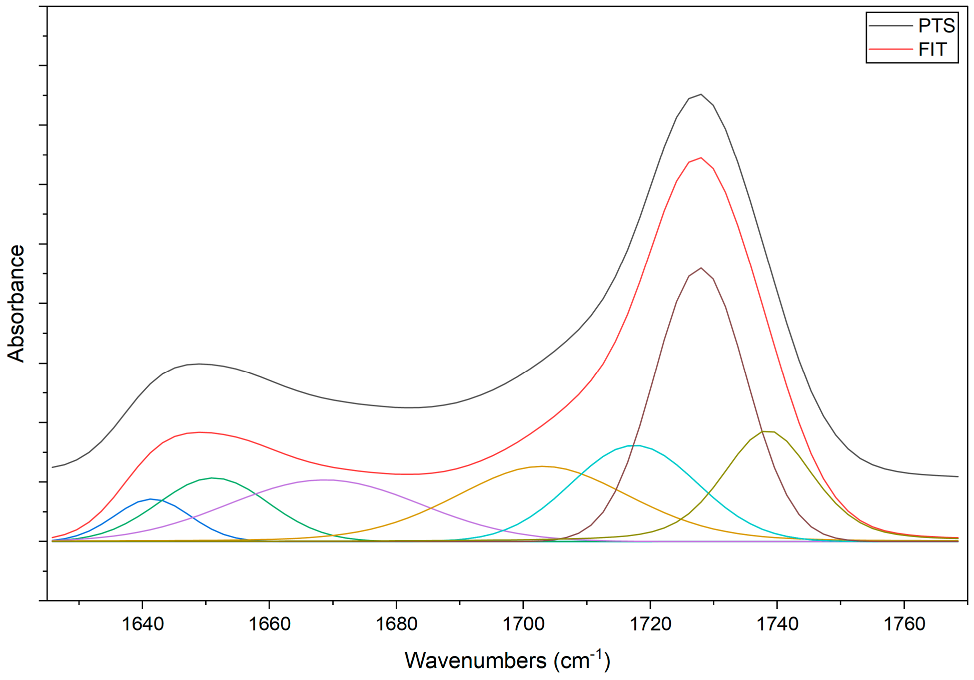

Figure S5. Deconvoluted spectrum of PTS sample.
